# Tobacco use among Australian dental hygiene students is declining, but more still needs to be done

**DOI:** 10.1186/1617-9625-11-22

**Published:** 2013-10-23

**Authors:** Melanie J Hayes, Derek R Smith

**Affiliations:** 1 School of Health Sciences, Faculty of Health and Medicine, University of Newcastle, Brush Road, 2258 Ourimbah, Australia

**Keywords:** Smoking, Tobacco, Students, Dental hygiene, Prevention

## Abstract

While health care professionals have a responsibility to prevent and control the use of tobacco for improved health outcomes, it appears that some dental hygiene students continue to smoke. A survey of Australian dental hygiene students found that up to 16.3% smoke, although this prevalence rate decreased with each year of study. As future role models, it is essential that smoking cessation counselling is embedded in the dental curriculum to not only discourage their own habits, but so that they may promote the importance of being tobacco free to the wider population.

## Findings

The World Health Organisation has identified a “world tobacco epidemic” and advised that unless urgent action is taken, over a billion people will die as a result of tobacco use in the 21st century [1]. Health professionals clearly have a duty to promote good health and tackle the “unmet challenge” of tobacco control; this includes educating the public about the harmful effects of smoking, and to provide preventive advice to assist smokers to quit [2].

As preventive therapists, dental hygienists are in a prime position to provide education about the damaging effects of tobacco and smoking cessation counselling to patients. Studies have revealed that most dental students believe that providing advice about smoking cessation is their responsibility as a health professional [3]. However, with this responsibility often comes the expectation to be a role model, and to practise what we preach; this begins as early as our dental education and training.

The smoking status of dental hygiene students at a regional University in Australia was explored via a series of cross-sectional surveys, as part of a larger study (Ethics Approval No. H-2008-0208). The overall project aimed at investigating the health and well-being of the cohort, and information on school study and exercise habits, and musculoskeletal pain was collected. A cohort of dental hygiene students was surveyed in each year of their program (2008–2010) and they were asked to identify their smoking status. In 2008, 7/43 students (16.3%) identified themselves as regular smokers; in 2009 7/44 (15.9%); and in 2010 5/36 (13.9%). The cohort was predominantly female.

When comparing to other health sciences students in Australia, the smoking rate among dental hygiene students appears to be fairly high. It has been previously reported, for example, that between 3-6% of medical students and 2-13% of dental students are regular smokers [2], while a study of occupational therapy students found no regular smokers at all [4]. Dental hygiene students in Australia have previously reported that 10.3% were smokers, which is also less than the current study [5].

The reported smoking status of dental hygiene students was however, less than had been identified in similar research conducted on dental hygiene students overseas. Dental hygiene students in Japan had reported that 20.3% were smokers [6]; 23.4% of students in the United States identified as smokers [7]; while smokers accounted for one-quarter (25%) of dental hygiene students in Ireland [8]. It is encouraging that the reported smoking status decreased throughout the three years of study; perhaps education regarding the detrimental effects of smoking on general and oral health influenced their decision to quit.

The above findings warrant the need to support dental hygiene students with the necessary training that would help them quit smoking themselves. Smoking cessation counselling should therefore be a compulsory part of dental hygiene education and training; as such dental hygiene students may be encouraged to cease smoking tobacco, and may influence others as both educators and role models in their chosen profession.

## Competing interests

The authors declare that they have no competing interests.

## Authors’ contributions

DRS conceived the idea for the study. MJH and DRS wrote the manuscript. Both authors read and approved the final version of the manuscript.
